# Prebiotic Effects of Xylooligosaccharides on the Improvement of Microbiota Balance in Human Subjects

**DOI:** 10.1155/2016/5789232

**Published:** 2016-08-29

**Authors:** Shyh-Hsiang Lin, Liang-Mao Chou, Yi-Wen Chien, Jung-Su Chang, Ching-I Lin

**Affiliations:** ^1^School of Nutrition and Health Sciences, Taipei Medical University, No. 250, Wuxing Street, Taipei City 11031, Taiwan; ^2^Department of Dietetics and Nutrition, Taipei Veterans General Hospital, No. 201, Sec. 2, Shipai Rd., Taipei City 11217, Taiwan; ^3^Department of Nutrition and Health Sciences, Kainan University, No. 1, Kainan Rd., Luzhu Dist., Taoyuan City 33857, Taiwan

## Abstract

It has been indicated that probiotics can be nourished by consuming prebiotics in order to function more efficiently, allowing the bacteria to stay within a healthy balance. In this study, we investigated the effects of xylooligosaccharides- (XOS-) enriched rice porridge consumption on the ecosystem in the intestinal tract of human subjects. Twenty healthy subjects participated in this 6-week trial, in which 10 subjects received XOS-enriched rice porridge while the others received placebo rice porridge. Fecal samples were collected at the end of weeks 0, 1, 3, 4, 6, and 7 for microorganism examination. The results showed that 6-week daily ingestion of the XOS-enriched rice porridge induced significant increases in fecal bacterial counts of* Lactobacillus* spp. and* Bifidobacterium* spp., as well as decreases in* Clostridium perfringens* without changing the total anaerobic bacterial counts, compared to that of placebo rice porridge. However, fluctuations in the counts of coliforms were observed in both groups during the 6-week intervention. In conclusion, the intestinal microbiota balance was improved after daily consumption of 150 g of rice porridge containing XOS for 6 weeks, demonstrating the prebiotic potential of XOS incorporated into foods. This also indicates the effectiveness of XOS as a functional ingredient in relation to its role as a prebiotic compound.

## 1. Introduction

It has been proposed that the dynamic and complex populations of gastrointestinal microorganisms play a pivotal role in human health [1, 2]. The intestinal microbiota not only exert metabolic activities but also participate in the defense against invading pathogens. It has been suggested that disruptions to intestinal microbial balance may lead to diseases including chronic intestinal diseases, colorectal cancer, type 2 diabetes, and obesity [1]. On the other hand, restoring the changed intestinal microbial balance to a more beneficial bacterial population may be beneficial in terms of supporting digestive or human health, which can be accomplished by administering probiotics and prebiotics or a combination of both (i.e., synbiotics) [2, 3].

Probiotics are bacteria that provide health-promoting properties for the host lining of the colon [2]. The most commonly used and/or studied probiotics are largely species of the genera* Bifidobacterium* and* Lactobacillus*, all of which can be found in the host's own microbiota and fermented foods [4]. Evidence suggests that these probiotic bacteria can alleviate lactose intolerance, inhibit the growth of harmful bacteria, prevent colon cancer, decrease cholesterol levels, improve digestion, reduce inflammation, and stimulate the immune system [4, 5]. Therefore, it has been considered that consuming probiotic rich foods (i.e., fermented foods) or supplements may replenish the beneficial bacterial populations, such as the genera* Bifidobacterium* and* Lactobacillus*, for the maintenance of gastrointestinal health or the prevention of diseases.

Prebiotics can nourish probiotics and encourage them to function more efficiently, allowing the bacteria to stay within a healthy balance [3]. They are nondigestible food ingredients, typically oligosaccharides that serve as the fuel for probiotics, allowing these beneficial microorganisms to thrive by going through the fermentation process [3]. Some of the commonly known prebiotics are fructooligosaccharides, galactooligosaccharides, and lactulose [6–8]. In addition, other types of oligosaccharides, such as isomaltooligosaccharides (IMO) and XOS, are emerging as a potential novel source of prebiotics that can be used as functional ingredients in foods [9, 10]. Of the emerging prebiotic oligosaccharides, XOS have attracted increasing interest because of their health, physicochemical, and technological related properties. XOS are mixtures of oligosaccharides containing *β*-1,4-linked xylose residues which naturally occur in bamboo shoots, fruits, vegetables, milk, and honey [11]. XOS has been found to be predominantly utilized by members of the* Bifidobacterium* genus [12]. Furthermore, the consumption of XOS results in increased indigenous* Bifidobacterium* spp. levels in the gastrointestinal tract and fecal short-chain fatty acids in rats [13, 14]. However, studies investigating the prebiotic effects of XOS on gastrointestinal microbiota in human populations have been limited. Thus, the objective of the present study was to examine the prebiotic effect of XOS incorporated into rice porridge on the ecosystem in the intestinal tract of human subjects.

## 2. Materials and Methods

### 2.1. Subjects

The project was approved by the Taipei Medical University-Joint Institutional Review Board, number 201209023 (TMU-JIRB201209023). Subjects were recruited from Taipei Medical University by advertising on noticeboards on campus. The general health status of all volunteers was assessed by the use of a standard medical questionnaire. Exclusion criteria for participation in the study were a history of gastrointestinal disease and chronic diseases. The subjects were asked to avoid consumption of antibiotics or any food/supplements that may influence the microbiota one week prior to and during the study period. Written informed consent was obtained from every subject before participating in the study. Subjects were instructed to maintain their usual dietary habits and normal lifestyles during the study while being assessed for the restriction of prebiotic consumption during a two-week period prior to the intervention. Baseline fecal samples were taken before the treatment period began.

### 2.2. Study Design and Treatments

**Figure 1:**
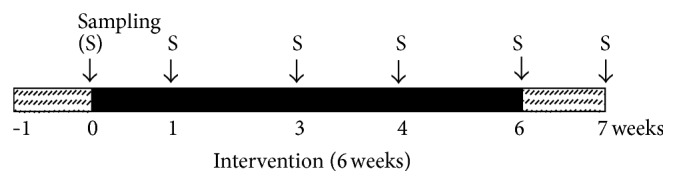
The experiment timetable.

A randomized, placebo-controlled study design was carried out with 20 subjects. The study comprised three phases: a 1-week run-in phase, a 6-week intervention phase, and a 1-week washout phase. During the 1-week run-in phase, all subjects were instructed to maintain their usual diet but to avoid consumption of other prebiotic and probiotic products during the experiment. Twenty subjects were randomly divided into two groups: XOS and placebo. During the 6-week intervention period, the XOS group (*n* = 10) was instructed to consume rice porridge containing XOS (150 g per package containing ~1.2 g of XOS), while the placebo group (*n* = 10) was instructed to consume rice porridge without XOS. The products consumed in both the XOS and the placebo groups were identical in appearance, taste, and color. The experimental and placebo products were consumed with breakfast once daily for six weeks. Fecal specimens from each subject were collected at the end of the run-in phase (week 0), the intervention phase (weeks 1, 3, 4, and 6), and the washout phase (week 7). Subjects were asked to keep a three-day dietary record (2 weekdays and 1 weekend) and a stool frequency and consistency record once a week throughout the whole experiment as a way of examining their adherence to the diet. The experiment timetable is shown in Figure 1.

### 2.3. Sample Collection and Microorganism Analyses

The middle section of a fecal sample from each subject was collected on the last day of weeks 0, 1, 3, 4, 6, and 7 and stored at −20°C for less than 24 h before analysis. Precisely 0.5 grams of the sample and 15 mL of anaerobic solution were thoroughly mixed to form a sample solution. Series of dilutions from 10^−1^ to 10^−6^ were prepared. Microorganism isolation and examination were performed using the methods previously developed [15]. In brief,* Bifidobacterium* spp. were incubated with bifidobacteria iodoacetate medium-25 for 48 hours (hr);* Lactobacillus* spp. were incubated with* Lactobacillus* anaerobic MRS with bromocresol green for 48 hr;* Clostridium perfringens* were incubated with tryptose-sulfite-D-cycloserine agar for 24-hr; coliform organisms were incubated with Endo agar plates for 24-hr; total anaerobic organisms were examined with CDC anaerobic blood agar. When counting colonies, plates with 30–300 colonies were included. The number of bacteria was presented as log CFU/g of wet weight of feces. The calculation formulae are listed as follows. For* Bifidobacterium* spp.,* Lactobacillus* spp., coliform organisms, and total anaerobic organisms, the formula is CFU/plate × 20 (50 *μ*L/plate) × dilution factor × 15 mL/sample (g), and, for* Clostridium perfringens*, the formula is CFU/plate × dilution factor × 15 mL/sample (g).

### 2.4. Statistical Analysis

Data are presented as the mean ± SD. Analysis of variance (ANOVA) with repeated measures and paired* t*-tests were performed using SAS version 9.1.* p* values smaller than 0.05 are considered statistically significant.

## 3. Results

All subjects successfully completed the experiment. The characteristics of the participants are shown in Table 1. No significant difference was observed in age (23.4 ± 1.6 versus 25.0 ± 1.7 years), BMI (19.7 ± 2.4 versus 20.7 ± 2.1 kg/m^2^), or sex distribution (2 men and 8 women versus 2 men and 8 women) between the XOS and placebo groups.

**Figure 2:**
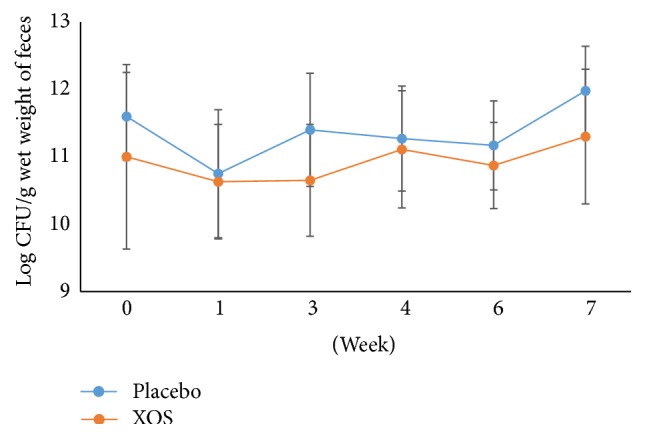
The changes in logarithm number (mean ± SD) of total anaerobic bacteria during the trial. Weeks 0-1: a run-in phase. Weeks 6-7: a 1-week washout phase.

**Figure 3:**
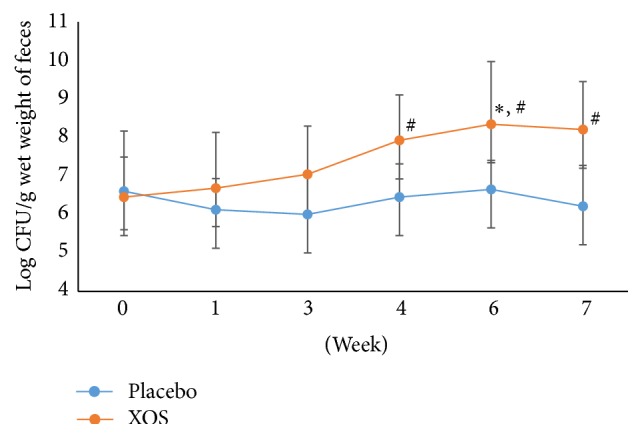
The changes in logarithm number (mean ± SD) of* Lactobacillus* spp. during the trial. Weeks 0-1: a run-in phase. Weeks 6-7: a 1-week washout phase. ^#^Repeated measures ANOVA on different groups across time. ^*∗*^Paired* t*-test of week 6 versus week 0 within a group. *p* < 0.05.

There were no significant differences in the amounts of total anaerobic bacteria,* Lactobacillus* spp.,* Bifidobacterium* spp., coliforms, and* Clostridium perfringens* in week 0 (the end of the run-in phase) between the two groups. After the 6-week intervention, as shown in Figure 2, the total anaerobic bacterial counts of the experimental and the placebo groups were not statistically different. In contrast, during the intervention period, the XOS group had significantly higher* Lactobacillus* spp. counts compared to the placebo group participants at weeks 4 and 6 (*p* < 0.05, Figure 3) and even after the 1-week washout period. In the XOS group, at week 6, the number of* Lactobacillus* spp. (Figure 3) was significantly higher than at week 0 (*p* < 0.05).

**Figure 4:**
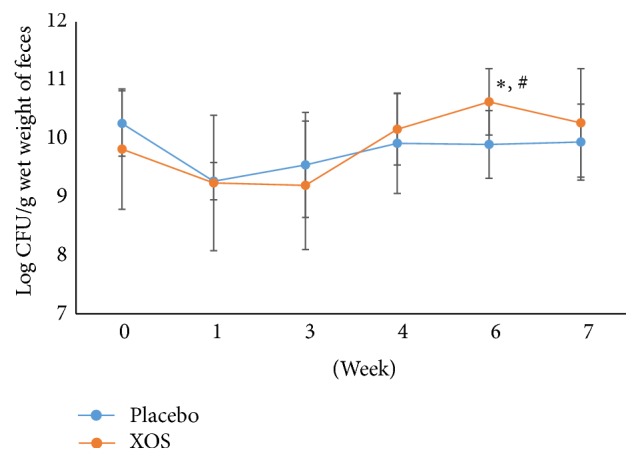
The changes in logarithm number (mean ± SD) of* Bifidobacterium* spp. during the trial. Weeks 0-1: a run-in phase. Weeks 6-7: a 1-week washout phase. ^#^Repeated measures ANOVA on different groups across time. ^*∗*^Paired* t*-test of week 6 versus week 0 within a group. *p* < 0.05.

**Figure 5:**
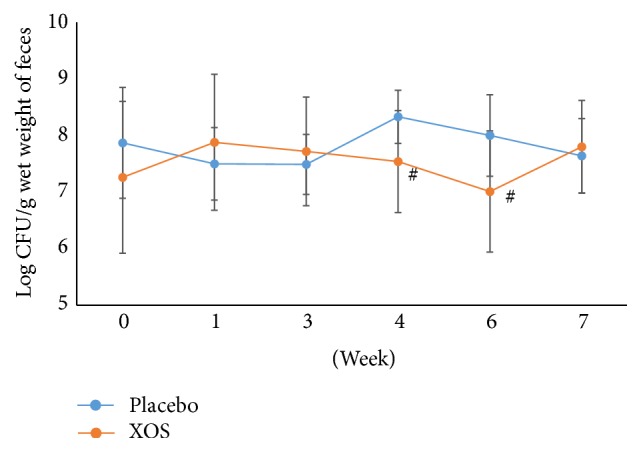
The changes in logarithm number (mean ± SD) of coliforms during the trial. Weeks 0-1: a run-in phase. Weeks 6-7: a 1-week washout phase. ^#^Repeated measures ANOVA on different groups across time. *p* < 0.05.

**Figure 6:**
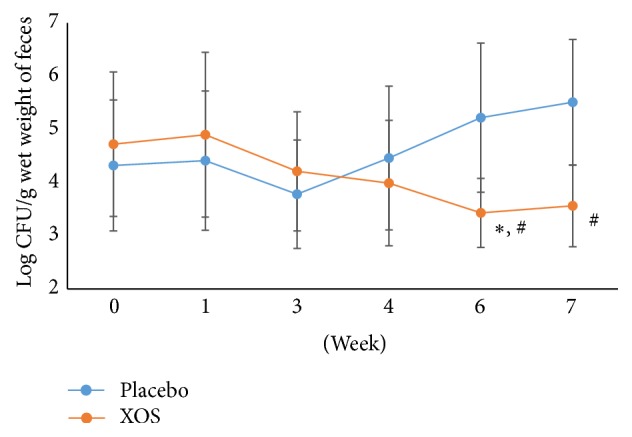
The changes in logarithm number (mean ± SD) of* Clostridium perfringens* during the trial. Weeks 0-1: a run-in phase; Weeks 6-7: a 1-week washout phase. ^#^Repeated measures ANOVA on different groups across time. ^*∗*^Paired* t*-test of week 6 versus week 0 within a group. *p* < 0.05.

*Bifidobacterium* spp. counts remained similar for the XOS and placebo groups during the study except for a significant increase in the XOS group (Figure 4) as compared to the placebo group at week 6 (*p* < 0.05) and the baseline (Figure 4). However, the XOS group showed no significant differences in coliform populations (at weeks 4 and 6) as compared with week 0 (Figure 5). Both groups showed fluctuations throughout the study. In addition, the placebo group demonstrated a trend toward an elevation in coliform populations at week 4 as compared to weeks 1 and 3. Compared to the placebo group, the XOS group had lower populations of* Clostridium perfringens* at week 6 and one week after (Figure 6). However, at week 3, the placebo group showed similar counts for* Clostridium perfringens* to the XOS group at weeks 6 and 7. Yet, fluctuations and sudden increases occurred in the placebo group for* Clostridium perfringens* counts at weeks 6 and 7 compared to week 3 as well as to week 0 (Figure 6).

## 4. Discussion

In this study, we conducted a randomized, controlled study with the aim of evaluating the prebiotic effects of XOS on fecal microbiota in healthy human volunteers after a period of 6 weeks of daily consumption since the prebiotic evidence derived from human trials is still insufficient. More than 1,000 microbial species are known to inhabit the human GI tract, constituting a complex ecological community, which is also referred to as the intestinal microbiota [16]. This ecosystem contains approximately 10^14^ microorganisms which are predominantly represented by anaerobic bacterial species [17], and the vast majority of these reside in the colon with the populations reaching densities of up to 10^12^ counts per gram of content [16]. Among these microbiota,* Lactobacillus* and* Bifidobacterium* species are beneficial due to their fermentation characteristics, whereas* Clostridium perfringens* is detrimental to health because it is an opportunistic pathogen with the capability of causing food poisoning and necrotic enteritis [18]. Studies have demonstrated that the intestinal microbiota are crucial to human health, and alterations in this ecosystem are linked to several diseases such as irritable bowel syndrome, type 2 diabetes, and obesity [1, 16]. Overall, the results suggest that changes in the bacterial growth were mediated by XOS incorporation into the food product. The addition of XOS to foodstuffs has prebiotic potential to alter the composition of the intestinal microbiota that could be relevant to intestinal health. In the present study, the counts of total anaerobic bacteria were unaffected by the addition of XOS to the rice porridge throughout the study, indicating that total anaerobic bacteria numbers remained steady instead of growing abnormally. Furthermore, we observed that consumption of rice porridge containing XOS resulted in a significant increase in the numbers of* Lactobacillus* spp. and* Bifidobacterium* spp. compared with the placebo, demonstrating the prebiotic potential of XOS as a food ingredient. It is well documented that both* Lactobacillus* spp. and* Bifidobacterium* spp. are preferentially able to ferment XOS, thereby utilizing them as an energy source for growth [11, 19, 20]. The prebiotic effect of XOS observed here, particularly the increased* Bifidobacterium* spp., is consistent with a previous intervention study, where capsule supplements containing XOS have been used [21]. However, regarding the observed changes in* Lactobacillus* spp., our result contradicted a previous study, which showed that the number of* Lactobacillus* spp. was unchanged after XOS supplementation [21]. These inconsistent results might reflect differences in the participants being studied or in the methodologies employed.

The increased populations of the health-promoting bacteria after prebiotic administration have been shown to eliminate the presence of pathogenic or potential pathogenic bacteria [7]. In the current study, the levels of fecal coliforms in response to treatments were also examined as this bacterial group is commonly used as an indicator of water contamination and the possible presence of pathogens [22]. However, fluctuating numbers of the fecal coliforms in both groups at weeks 4 and 6 were observed. It was difficult to determine whether these changes were attributed to the dietary intervention (e.g., the rice-based porridge) or other dietary factors, as all participants were allowed to consume their usual diets. Nevertheless, the observed lower number of fecal coliforms in the XOS group suggests that the XOS-containing diet seemed to be better tolerated. Furthermore, a similar response was observed, in which the abundance of pathogenic bacteria,* Clostridium perfringens*, was significantly lower in the fecal samples of the XOS group than in those of the control group. These data could be explained by the XOS suppressing the growth of* Clostridium perfringens*; the mechanisms underlying this effect are likely due to the production of short-chain fatty acids (SCFAs) via the fermentation of XOS in the colon [13, 23]. A decrease in intestinal pH has been reported as a consequence of the increased SCFA production which subsequently inhibits the overgrowth of pathogenic bacteria [24]. However, it should be noted that the* Clostridium perfringens* counts exhibited a modest upward trend following the placebo treatment in the present study. From this finding, the possibility that rice porridge itself manipulates the growth of* Clostridium perfringens* cannot be ruled out. Previous studies have shown that polished rice ingestion appears to increase the number of fecal pathogenic bacteria, such as* Clostridium perfringens*, compared to unpolished rice [25]. In other words, this suggests that the presence of rice fiber may have the ability to depress the growth of certain intestinal pathogenic bacteria [25]. Additionally, there are some limitations to this study which should be noted. Firstly, the power of this investigation can be increased as the total number of subjects is increased. Secondly, fiber in the diet can have an impact on gut microbiota [26]; however, although we did not accurately measure the total intake of dietary fiber for the comparison between groups, we randomly assigned our subjects into the groups and reminded them not to change their dietary habits. Thus, the amount of fiber ingested should not be an issue in this study.

In conclusion, the intestinal bacterial phase was improved after the daily consumption of 150 g of rice porridge containing XOS for 6 weeks, suggesting the beneficial effects of XOS on intestinal functions and health. Further research on the incorporation of XOS into other kinds of food is warranted.

## Figures and Tables

**Table 1:** Basic characteristics of subjects.

	*n*	Age (years)	Height (cm)	Weight (kg)	BMI (kg/m^2^)
Placebo					
Male	2	24.5 ± 1.5	170.5 ± 2.7	68.5 ± 1.0	23.3 ± 0.6
Female	8	25.2 ± 1.8	160.8 ± 4.6	51.7 ± 5.5	20.0 ± 1.5

Total	10	25.0 ± 1.7	162.7 ± 5.8	55.1 ± 8.6	20.7 ± 2.1

XOS					
Male	2	24.5 ± 3.5	173.5 ± 2.1	67.0 ± 0.0	22.3 ± 0.5
Female	8	23.1 ± 1.0	159.6 ± 5.7	48.4 ± 4.1	19.1 ± 2.3

Total	10	23.4 ± 1.6	162.4 ± 7.7	52.1 ± 8.7	19.7 ± 2.4

## References

[1] Guinane C. M., Cotter P. D. (2013). Role of the gut microbiota in health and chronic gastrointestinal disease: understanding a hidden metabolic organ. *Therapeutic Advances in Gastroenterology*.

[2] Fijan S. (2014). Microorganisms with claimed probiotic properties: an overview of recent literature. *International Journal of Environmental Research and Public Health*.

[3] Gibson G. R., Probert H. M., Van Loo J., Rastall R. A., Roberfroid M. B. (2004). Dietary modulation of the human colonic microbiota: updating the concept of prebiotics. *Nutrition Research Reviews*.

[4] Parvez S., Malik K. A., Ah Kang S., Kim H.-Y. (2006). Probiotics and their fermented food products are beneficial for health. *Journal of Applied Microbiology*.

[5] Farnworth E. R. (2008). The evidence to support health claims for probiotics. *The Journal of Nutrition*.

[6] Sabater-Molina M., Larqué E., Torrella F., Zamora S. (2009). Dietary fructooligosaccharides and potential benefits on health. *Journal of Physiology and Biochemistry*.

[7] Gibson G. R., Roberfroid M. B. (1995). Dietary modulation of the human colonic microbiota: introducing the concept of prebiotics. *The Journal of Nutrition*.

[8] Macgillivray P. C., Finlay H. V., Binns T. B. (1959). Use of lactulose to create a preponderance of Lactobacilli in the intestine of bottle-fed infants. *Scottish Medical Journal*.

[9] Gilad O., Jacobsen S., Stuer-Lauridsen B., Pedersen M. B., Garrigues C., Svensson B. (2010). Combined transcriptome and proteome analysis of *Bifidobacterium animalis* subsp. *lactis* BB-12 grown on xylo-oligosaccharides and a model of their utilization. *Applied and Environmental Microbiology*.

[10] Chung C.-H., Day D. F. (2004). Efficacy of Leuconostoc mesenteroides (ATCC 13146) isomaltooligosaccharides as a poultry prebiotic. *Poultry Science*.

[11] Vázquez M. J., Alonso J. L., Domínguez H., Parajó J. C. (2000). Xylooligosaccharides: manufacture and applications. *Trends in Food Science & Technology*.

[12] Amaretti A., Bernardi T., Leonardi A., Raimondi S., Zanoni S., Rossi M. (2013). Fermentation of xylo-oligosaccharides by *Bifidobacterium adolescentis* DSMZ 18350: kinetics, metabolism, and *β*-xylosidase activities. *Applied Microbiology and Biotechnology*.

[13] Campbell J. M., Fahey G. C., Wolf B. W. (1997). Selected indigestible oligosaccharides affect large bowel mass, cecal and fecal short-chain fatty acids, pH and microflora in rats. *The Journal of Nutrition*.

[14] Hsu C.-K., Liao J.-W., Chung Y.-C., Hsieh C.-P., Chan Y.-C. (2004). Xylooligosaccharides and fructooligosaccharides affect the intestinal microbiota and precancerous colonic lesion development in rats. *The Journal of Nutrition*.

[15] Cheng I.-C., Shang H.-F., Lin T.-F., Wang T.-H., Lin H.-S., Lin S.-H. (2005). Effect of fermented soy milk on the intestinal bacterial ecosystem. *World Journal of Gastroenterology*.

[16] Sekirov I., Russell S. L., Antunes L. C. M., Finlay B. B. (2010). Gut microbiota in health and disease. *Physiological Reviews*.

[17] Harris M. A., Reddy C. A., Carter G. R. (1976). Anaerobic bacteria from the large intestine of mice. *Applied and Environmental Microbiology*.

[18] Uzal F. A., McClane B. A., Cheung J. K. (2015). Animal models to study the pathogenesis of human and animal *Clostridium perfringens* infections. *Veterinary Microbiology*.

[19] Pan X., Wu T., Zhang L., Cai L., Song Z. (2009). Influence of oligosaccharides on the growth and tolerance capacity of lactobacilli to simulated stress environment. *Letters in Applied Microbiology*.

[20] Rycroft C. E., Jones M. R., Gibson G. R., Rastall R. A. (2001). A comparative in vitro evaluation of the fermentation properties of prebiotic oligosaccharides. *Journal of Applied Microbiology*.

[21] Finegold S. M., Li Z., Summanen P. H. (2014). Xylooligosaccharide increases bifidobacteria but not lactobacilli in human gut microbiota. *Food & Function*.

[22] Templar H. A., Dila D. K., Bootsma M. J., Corsi S. R., McLellan S. L. (2016). Quantification of human-associated fecal indicators reveal sewage from urban watersheds as a source of pollution to Lake Michigan. *Water Research*.

[23] Okazaki M., Fujikawa S., Matsumoto N. (1990). Effect of xylooligosaccharide on the growth of bifidobacteria. *Bifidobacteria and Microflora*.

[24] Duncan S. H., Louis P., Thomson J. M., Flint H. J. (2009). The role of pH in determining the species composition of the human colonic microbiota. *Environmental Microbiology*.

[25] Benno Y., Endo K., Miyoshi H., Okuda T., Koishi H., Mitsuoka T. (1989). Effect of rice fiber on human fecal microflora. *Microbiology and Immunology*.

[26] Slavin J. (2013). Fiber and prebiotics: mechanisms and health benefits. *Nutrients*.

